# Roll-to-roll sputtered and patterned Cu_2−*x*_O/Cu/Cu_2−*x*_O multilayer grid electrode for flexible smart windows†

**DOI:** 10.1039/c8ra03252a

**Published:** 2018-07-30

**Authors:** Hyeong-Jin Seo, Yoon-Chae Nah, Han-Ki Kim

**Affiliations:** School of Advanced Materials Science & Engineering, Sungkyunkwan University 2066 Seobu-ro Jangan-gu, Suwon Gyeonggi-do 440-746 Republic of Korea hankikim@skku.edu +82-31-290-7410 +82-31-290-7390; IPCE, Dept. of Energy, Materials, and Chemical Engineering, Korea University of Technology and Education Cheonan 31253 Republic of Korea

## Abstract

We fabricated cost-effective Cu_2−*x*_O/Cu/Cu_2−*x*_O multilayer grid electrodes using roll-to-roll (RTR) sputtering and patterning processes for use as transparent and flexible electrodes in flexible smart windows. To optimize the patterned Cu_2−*x*_O/Cu/Cu_2−*x*_O multilayer grid, the electrical and optical properties of the Cu_2−*x*_O/Cu/Cu_2−*x*_O multilayer grid electrodes were investigated as a function of grid width and pitch, which directly influence the filling factor of the grid. At the optimized grid width of 16 and pitch of 600 μm, the Cu_2−*x*_O/Cu/Cu_2−*x*_O multilayer grid had a sheet resistance of 7.17 Ohm per square and an optical transmittance of 87.6%. In addition, the mechanical properties of the optimized Cu_2−*x*_O/Cu/Cu_2−*x*_O multilayer grid electrode was compared to those of brittle ITO electrodes to demonstrate its outstanding flexibility. To show the potential of the Cu_2−*x*_O/Cu/Cu_2−*x*_O multilayer grid for smart windows, we fabricated a flexible and transparent thin film heater (TFH) and a flexible electrochromic (EC) device, which are key components of smart windows. The low saturation voltage of the Cu_2−*x*_O/Cu/Cu_2−*x*_O grid-based TFH and the fast on–off performance of the Cu_2−*x*_O/Cu/Cu_2−*x*_O grid-based EC device indicates that the RTR-processed Cu_2−*x*_O/Cu/Cu_2−*x*_O multilayer grid is promising as a low-cost and large-area flexible transparent electrode for high-performance smart windows.

## Abstract 2

We fabricated cost-effective Cu_2−*x*_O/Cu/Cu_2−*x*_O multilayer grid electrodes using roll-to-roll (RTR) sputtering and patterning processes for use as transparent and flexible electrodes in flexible smart windows.

## Graphical abstract

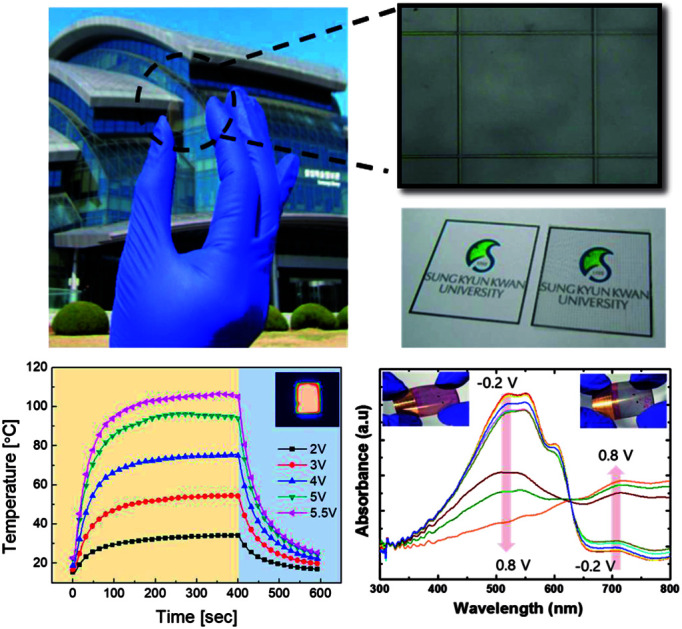

## 1. Introduction

Multi-functional smart windows equipped with transparent displays, transparent heaters, electrochromic devices, and energy-harvesting devices have attracted significant attention as next-generation exterior materials for buildings and automobiles.^1–5^ Unlike conventional windows, which simply pass visible light into the building or automobile, smart windows provide several convenient and smart functions, such as information displays, energy harvesting, self-heating and cleaning, transmittance control, and indoor temperature and light control. In several components of smart windows, flexible and transparent thin film heaters (TFHs) can remove frost or ice by heating the window and flexible electrochromic (EC) devices, which can adjust the indoor brightness by controlling the transmittance of the window.^5–7^ The performance, stability, and fabrication cost of TFHs and EC devices are critically dependent on the electrical, optical, and mechanical properties of transparent and flexible electrodes (TFEs). In addition, the fabrication cost of TFHs and EC devices for low-cost and large-area smart windows is closely related to the cost of the TFE materials and coating processes. Therefore, the development of highly transparent, conductive, flexible, and cost-effective TFE materials and processing methods is imperative for mass-producing TFHs and EC devices. A common transparent conducting electrode (TCE) material, Sn-doped In_2_O_3_ (ITO) films coated on polyethylene terephthalate (PET) substrates are typically employed as TFEs due to their low sheet resistance, high optical transmittance, well-known processing technology, and ease of use for large-area coatings.^8–12^ However, sputtered ITO films are critically limited as high-quality and cost-effective TFEs due to the relatively high sheet resistance and poor mechanical properties of ITO/PET films as well as the high cost of indium.^13^ Several TCE materials fabricated by vacuum-based or solution-based coating processes have been extensively reported as replacements for high-cost ITO films.^14–19^ Among these, sputtered oxide–metal–oxide (OMO) multilayer films and printed metal (Ag, Cu) grid electrodes are considered to be promising replacements. However, sputtered OMO electrodes are still composed of high-cost indium-based oxide and Ag interlayers. In the case of the printed metal grid electrodes, the patterned grid is shiny due to the high reflection on the surface. To solve both of these problems, it is necessary to develop a cost-effective OMO-based multilayer grid electrode that combines the merits of the OMO and the metal grid.

In this work, we developed a grid-patterned OMO multilayer electrode with a Cu_2−*x*_O/Cu/Cu_2−*x*_O structure using a lab-scale roll-to-roll (RTR) sputtering system to replace high-cost ITO electrodes. To optimize the grid width and pitch of the Cu_2−*x*_O/Cu/Cu_2−*x*_O multilayer grid, we investigated the electrical and optical properties of the Cu_2−*x*_O/Cu/Cu_2−*x*_O multilayer grid as a function of grid width and pitch. In addition, the mechanical properties of the Cu_2−*x*_O/Cu/Cu_2−*x*_O multilayer as a substitute for a typical sputtered ITO electrode were comprehensively investigated using lab-designed outer and inner bending tests. Furthermore, we used our patterned OMO multilayer grid electrode to fabricate flexible TFHs and EC devices to demonstrate their feasibility for application in next-generation flexible smart windows.

## 2. Experimental

### 2.1 Continuous roll-to-roll sputtering and patterning of Cu_2−*x*_O/Cu/Cu_2−*x*_O multilayer grid on a PET substrate

**Fig. 1:**
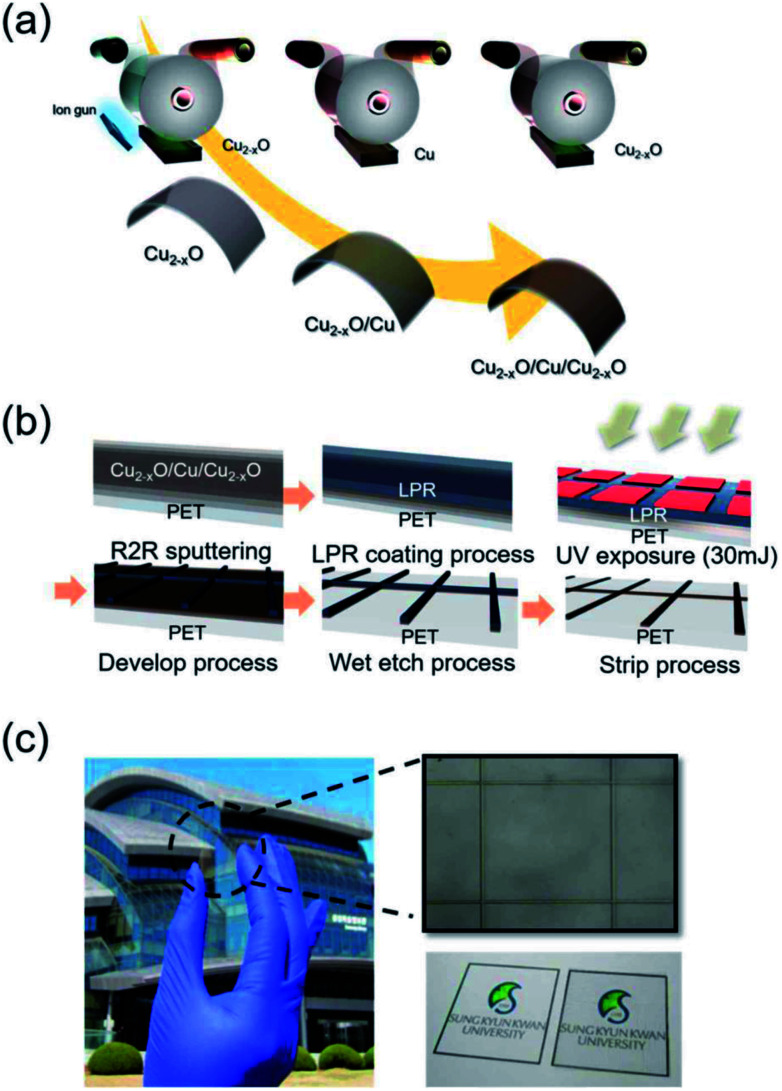
(a) Schematics of roll-to-roll sputtering and (b) roll-to-roll patterning of the Cu_2−*x*_O/Cu/Cu_2−*x*_O multilayer grid prepared on a PET substrate. (c) Picture of the grid-patterned Cu_2−*x*_O/Cu/Cu_2−*x*_O multilayer and optical microscope image of the patterned grid electrode.

A Cu_2−*x*_O/Cu/Cu_2−*x*_O multilayer was deposited on a 125 μm-thick PET substrate (Kimoto Ltd., Japan) using a lab-scale RTR sputtering system with a linear ion gun and linear cathodes. Using a Cu rectangular target with a size of 460 mm × 130 mm, the Cu-deficient Cu_2−*x*_O layer was deposited by reactive sputtering in an Ar/O_2_ ambient atmosphere and the Cu layer was deposited by DC sputtering in a pure Ar ambient atmosphere in the same RTR chamber. In detail, a 150 nm-thick bottom Cu_2−*x*_O layer was directly sputtered on the PET substrate after treating it with an Ar beam. The Cu layer was reactively sputtered on the PET substrate at a constant rolling speed of 0.4 m min^−1^ by applying a DC power of 2.2 kW to a Cu target under Ar/O_2_ gas with a flow rate of 400/120 sccm. A 150 nm-thick Cu interlayer was then sputtered on the bottom Cu_2−*x*_O layer at a DC power of 2.2 kW under a pure Ar gas flow of 450 sccm. Finally, the top Cu_2−*x*_O layer was reactively sputtered on the Cu metal interlayer under the same sputtering conditions as the bottom Cu_2−*x*_O layer. Fig. 1(a) shows a schematic of the RTR sputtering process for the Cu_2−*x*_O/Cu/Cu_2−*x*_O multilayer. Due to the semi-transparency of the Cu_2−*x*_O layer and the opacity of the Cu layer, the as-deposited Cu_2−*x*_O/Cu/Cu_2−*x*_O multilayer had a dark brown color with an optical transmittance of 0% (ESI Fig. S1†). To pattern the opaque Cu_2−*x*_O/Cu/Cu_2−*x*_O multilayer, a liquid photoresist (LPR) layer was coated on the sputtered Cu_2−*x*_O/Cu/Cu_2−*x*_O multilayer using a commercial slot die coating system. The LPR-coated Cu_2−*x*_O/Cu/Cu_2−*x*_O multilayer was passed to a heating chamber by means of an unwinding and rewinding roller. The Cu_2−*x*_O/Cu/Cu_2−*x*_O multilayer was exposed to ultraviolet (UV) light using a positive grid mask with varying grid width and pitch. After UV irradiation, the Cu_2−*x*_O/Cu/Cu_2−*x*_O multilayer was patterned using a developing solution (DN-DT238E). Then, the photolithographed Cu_2−*x*_O/Cu/Cu_2−*x*_O multilayer was wet-etched using an etching solution (0.5% FeCl_3_ in deionized water) to form the grid structure. The wet-etched Cu_2−*x*_O/Cu/Cu_2−*x*_O multilayer was then stripped using a stripping solution. The stripped Cu_2−*x*_O/Cu/Cu_2−*x*_O multilayer grid was rinsed with deionized water. Fig. 1(b) shows the roll-to-roll patterning process for the Cu_2−*x*_O/Cu/Cu_2−*x*_O multilayer grid. The patterned Cu_2−*x*_O/Cu/Cu_2−*x*_O grid had high optical transmittance due to the narrow grid width and wide pitch, as shown in Fig. 1(c), unlike the as-deposited Cu_2−*x*_O/Cu/Cu_2−*x*_O films with dark brown color. The electrical and optical properties of the Cu_2−*x*_O/Cu/Cu_2−*x*_O multilayer grid electrode were investigated as a function of grid width and pitch using Hall measurements (HL5500PC, Accent Optical Technology) and a UV/visible spectrometer (UV 540, Unicam). In addition, the composition and binding energy of the reactive sputtered Cu_2−*x*_O film in the multilayer grid electrode were analyzed by using X-ray photoelectron spectroscopy (XPS: ESCALAB250). The mechanical properties of the grid-patterned Cu_2−*x*_O/Cu/Cu_2−*x*_O multilayer were evaluated using a specially designed inner and outer bending system. In addition, a dynamic fatigue bending test was performed using a lab-designed cyclic bending system operating at 0.5 Hz for 10 000 cycles.

### 2.2 Fabrication and evaluation of thin film heaters and electrochromic devices

To demonstrate the potential of the grid-patterned Cu_2−*x*_O/Cu/Cu_2−*x*_O multilayer, we fabricated flexible TFHs and EC devices on an optimized Cu_2−*x*_O/Cu/Cu_2−*x*_O multilayer grid electrode. Flexible TFHs with size of 25 × 25 mm^2^ with two-terminal side contacts was fabricated on the Cu_2−*x*_O/Cu/Cu_2−*x*_O multilayer grid (Fig. S2†). A 200 nm-thick Ag side contact electrode was sputtered onto the edge of the grid, and a DC voltage was supplied by a power supply (OPS 3010, ODA Technologies) to the grid-based TFHs through the Ag contact electrode at the film edge. The temperature of the TFHs was measured using a thermocouple mounted on their surface and an infrared (IR) thermal imager (A35sc, FLIR). Fig. S3† shows a picture of the temperature measurement system, including the thermocouple and IR thermal imager. A commercial ITO electrode with sheet resistance of 38.27 Ohm per square and optical transmittance of 87.52% was used as a reference. We also fabricated flexible electrochromic devices on the optimized Cu_2−*x*_O/Cu/Cu_2−*x*_O multilayer grid electrode. Prior to the deposition of poly(3-hexylthiophene) (P3HT), a poly(3,4-ethylenedioxythiophene):polystyrene sulfonate (PEDOT:PSS) film (NanoWearable Co.) was spin-coated at 1500 rpm for 30 s and cured at 120 °C for 5 min. A 2.2 wt% solution of P3HT (Aldrich) in chlorobenzene (Aldrich) was spin-coated on the PEDOT:PSS/grid substrates at 1500 rpm for 20 s and then dried on a hot plate at 60 °C for 10 min. The thickness of the P3HT films was around 60 nm, as measured using a fused ion beam-scanning electron microscope (FIB-SEM) (Helios NanoLab 600i). Electrochromic tests were performed in a three-electrode system with a propylene carbonate solution containing 0.5 M LiClO_4_. The working electrode was the P3HT film on a grid electrode. Pt wire and Ag/Ag^+^ wire were used as the counter and reference electrodes, respectively. The potential of the samples was controlled using a potentiostat/galvanostat (PGSTAT 302N, Autolab) and the optical properties of P3HT were measured using a UV/vis spectrometer (Cary 100, Agilent Technologies). The pulse potential tests were carried out by applying −0.2 V for coloring and 0.8 V for bleaching. Each coloring and bleaching time was set to 60 s.

## 3. Results and discussion

**Fig. 2:**
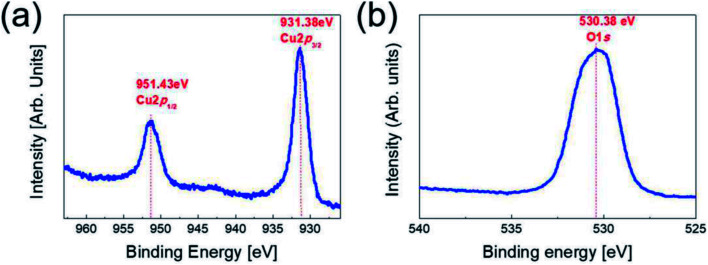
Core level spectra of (a) the Cu 2p peaks (b) the O 1s peak obtained from top Cu_2−*x*_O layer in the Cu_2−*x*_O/Cu/Cu_2−*x*_O multilayer grid.

To investigate the stoichiometry and phase of the copper oxide fabricated by RTR sputtering, we carried out XPS analysis for the top Cu_2_O layer in the Cu_2−*x*_O/Cu/Cu_2−*x*_O multilayer grid. Fig. 2 shows the XPS core level spectra of the Cu 2p peaks and the O 1s peaks obtained from Cu_2−*x*_O/Cu/Cu_2−*x*_O grid multilayer film. In general, reactive sputtered Cu could form two different oxides, such as cuprous oxide (Cu_2_O) and cupric oxide (CuO), depending on the oxygen flow ratio.^20,21^ The binding energies of the Cu 2p_1/2_(951.43 eV) and 2p_3/2_ (931.38 eV) were matched with general cuprous Cu_2_O phase.^22,23^ The O 1s peak at 530.38 eV was also matched with cuprous Cu_2_O phase.^24^ In addition, XPS analysis showed that the RTR sputtered Cu_2_O phase had a Cu-deficiency (Cu/O ratio: 1.88–1.92). Therefore, we concluded that the multilayer grid was consisted of top and bottom Cu_2−*x*_O phase. In our previous work, we also confirmed the cuprous Cu_2_O phase of the reactive RTR sputtered copper oxide films using XRD and TEM examinations.^23^

Thickness of each layer in the Cu_2−*x*_O/Cu/Cu_2−*x*_O multilayer grid electrodes was determined by consideration of optical transmittance, conductivity and adhesion of the multilayer grid. Generally, the high reflection from Cu metal interlayer resulted in glittering of the Cu grid, which prevent the use of Cu grid electrode for large area smart window. However, covering of 150 nm thick black Cu_2−*x*_O layer on the Cu interlayer could effectively reduce the reflection from Cu layer. Due to existence of the thick Cu_2−*x*_O layer led to no glittering from the Cu_2−*x*_O/Cu/Cu_2−*x*_O multilayer grids. In case of 150 nm thick bottom Cu_2−*x*_O layer, it improved adhesion of the Cu_2−*x*_O/Cu/Cu_2−*x*_O multilayer grid with PET substrate. Because reactive sputtered Cu_2−*x*_O layer sandwiched the Cu metal interlayer has a semiconducting property, the thick Cu metallic layer is necessary in the multilayer grid electrode for obtaining a low sheet resistance comparable to typical metal grid electrodes.

**Fig. 3:**
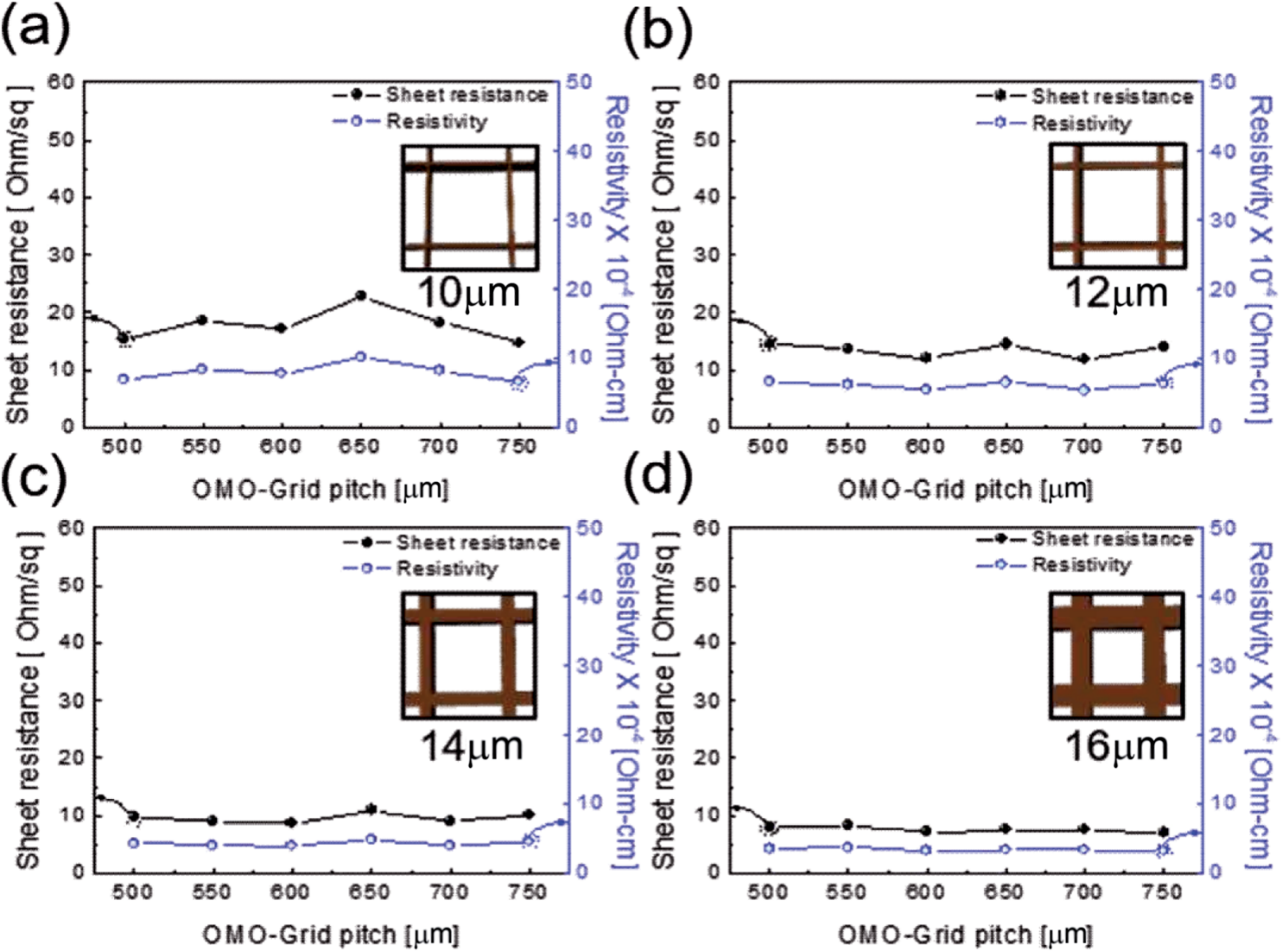
Sheet resistance and resistivity of the Cu_2−*x*_O/Cu/Cu_2−*x*_O multilayer grid electrode as a function of pitch length at a constant grid width of (a) 10, (b) 12, (c) 14, and (d) 16 μm. The inset shows the constant grid width.

Fig. 3 shows the sheet resistance and resistivity of the patterned Cu_2−*x*_O/Cu/Cu_2−*x*_O multilayer grid electrodes as a function of grid line width and pitch. In general, the electrical and optical properties of metal grid electrodes are critically dependent on the geometry of the grid, such as the grid width, grid height, and pitch.^25–27^ Therefore, it is very important to optimize the grid width and pitch to obtain low sheet resistance and high transmittance. Fig. 3(a)–(d) show the sheet resistance and resistivity of the Cu_2−*x*_O/Cu/Cu_2−*x*_O multilayer grid electrodes at a specific grid width (10, 12, 14, and 16 μm) with increasing pitch length. Each inset shows the geometry of the constant grid width. It is clear that the sheet resistance and resistivity of the Cu_2−*x*_O/Cu/Cu_2−*x*_O multilayer grid electrode decreased with increasing grid width from 10 to 16 μm. However, the grid pitch did not affect the sheet resistance or resistivity; with increasing grid pitch length from 500 to 750 μm, the grid electrode showed similar sheet resistance and resistivity because the changes in grid pitch were small. At a grid width of 16 μm, the Cu_2−*x*_O/Cu/Cu_2−*x*_O multilayer grid showed the lowest sheet resistance of 7.69 ± 0.59 Ohm per square and resistivity of 3.46 ± 0.2 × 10^−4^ Ohm cm^−1^. Due to the existence of the conductive Cu interlayer, the Cu_2−*x*_O/Cu/Cu_2−*x*_O multilayer grid exhibited better metallic conductivity than a typical ITO electrode. The calculated resistivity of the Cu interlayer was found to be 3.0 × 10^−6^ Ohm cm^−1^, which is similar to that of bulk Cu (1.7 × 10^−6^ Ohm cm^−1^).^23^ Therefore, the grid width of the Cu interlayer affected the electrical properties of the Cu_2−*x*_O/Cu/Cu_2−*x*_O multilayer grid because it provides the main current path.

**Fig. 4:**
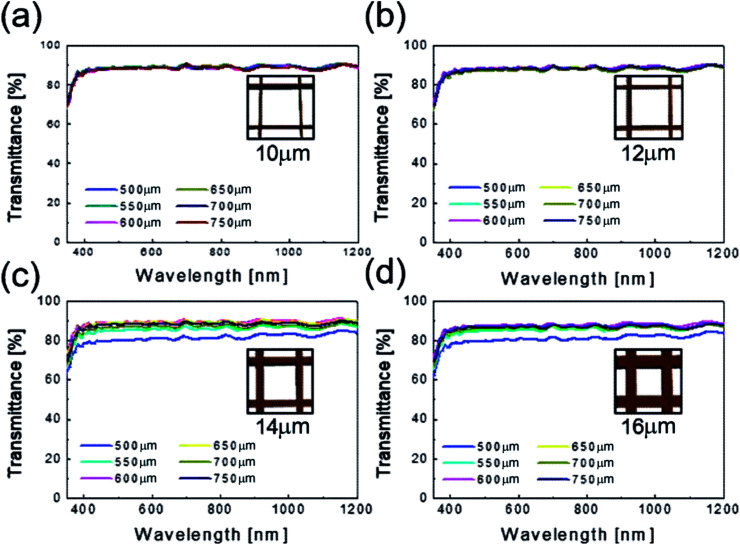
Optical transmittance of the Cu_2−*x*_O/Cu/Cu_2−*x*_O multilayer grid electrodes as a function of pitch length at a constant grid width of (a) 10, (b) 12, (c) 14, and (d) 16 μm.

Fig. 4 shows the optical transmittance of the Cu_2−*x*_O/Cu/Cu_2−*x*_O multilayer grid electrode as a function of grid width and pitch length. With increasing grid width and decreasing pitch length, the optical transmittance decreased, as shown in Fig. 4(a)–(d). At grid widths of 10 and 12 μm, the optical transmittance of the Cu_2−*x*_O/Cu/Cu_2−*x*_O multilayer grid electrode was almost constant regardless of grid pitch between 500 and 750 μm, as shown in Fig. 4(a) and (b). However, at grid widths of 14 and 16 μm, the optical transmittance of the multilayer grid was affected by pitch length. The optical transmittance of the electrode began to decrease at a grid pitch of 550 μm. Therefore, to obtain a high-performance multilayer grid electrode, the grid pitch should be larger than 550 μm. In the Cu_2−*x*_O/Cu/Cu_2−*x*_O multilayer grid electrodes, a high conductivity was determined by width of the metallic Cu layer because top and bottom Cu_2−*x*_O layers had semiconducting properties unlike typical OMO electrodes where the oxide layer is highly conductive oxide layer. In addition, the optical transmittance of the Cu_2−*x*_O/Cu/Cu_2−*x*_O grid electrode was dependent on the uncovered space in the grid structure because the Cu_2−*x*_O/Cu/Cu_2−*x*_O multilayer had an optical transmittance of 0% (Fig. S1†). Therefore, appropriate design of grid structure and geometry is very important to obtain high-quality multilayer grid electrodes.

**Fig. 5:**
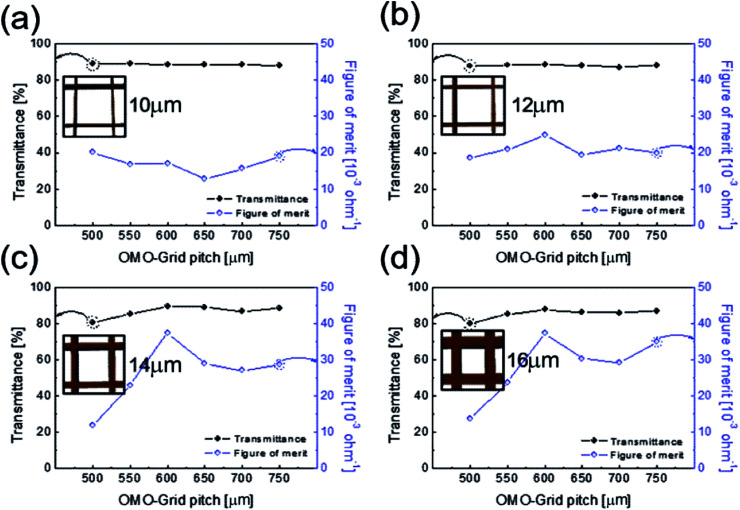
Figure of merit of the Cu_2−*x*_O/Cu/Cu_2−*x*_O multilayer grid electrode, calculated from the sheet resistance and optical transmittance at a 550 nm wavelength. The figure of merit is given as a function of pitch length at constant grid widths of (a) 10, (b) 12, (c) 14, and (d) 16 μm.

**Fig. 6:**
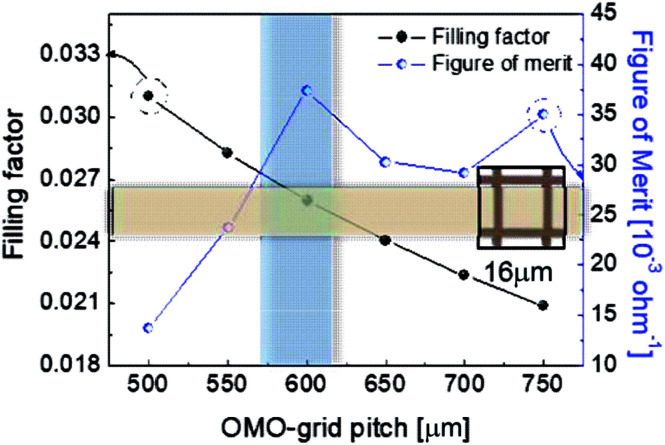
Filling factor and FOM of the Cu_2−*x*_O/Cu/Cu_2−*x*_O multilayer grid with a grid width of 16 μm as a function of grid pitch from 500 to 750 μm.

To determine the optimum grid width and pitch length of the Cu_2−*x*_O/Cu/Cu_2−*x*_O multilayer grid electrodes for flexible smart windows, the figure of merit (FOM; *T*^10^/*R*_sh_), as defined by Haacke,^28^ was evaluated from the measured sheet resistance (*R*_sh_) and optical transmittance (*T*) at a wavelength of 550 nm. As shown in Fig. 5(a) and (b), for very thin grid widths below 12 μm, the Cu_2−*x*_O/Cu/Cu_2−*x*_O multilayer grid electrode had a similar FOM value regardless of grid pitch length. The 12 μm grid showed slightly increased FOM values compared to the 10 μm grid due to the slightly decreased sheet resistance. However, at grid pitches of 14 and 16 μm, the FOM of the Cu_2−*x*_O/Cu/Cu_2−*x*_O multilayer grid was affected by grid pitch, as shown in Fig. 5(c) and (d). Due to the low optical transmittance, the grid had a low FOM at grid pitch lengths of 500 and 550 μm. The Cu_2−*x*_O/Cu/Cu_2−*x*_O multilayer with a grid width of 16 μm had the highest FOM (37.34 × 10^−3^ Ohm^−1^) at a grid pitch length of 600 μm. The grid width and pitch length dependence can be explained by the filling factor (*f*_F_) of the Cu_2−*x*_O/Cu/Cu_2−*x*_O multilayer grid. We calculated *f*_F_ as follows. Ghosh *et al.* reported that a Ni grid electrode has low sheet resistance and high optical transmittance at a fill factor of 0.025.^27^
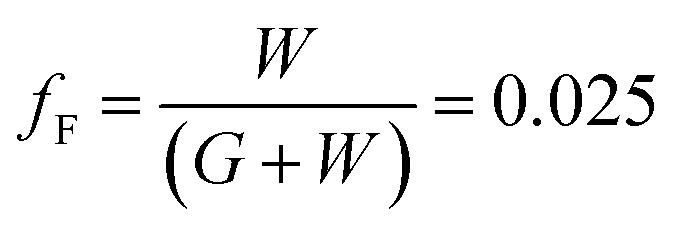
here, *W* and *G* are the widths and grid pitch of the Cu_2−*x*_O/Cu/Cu_2−*x*_O multilayer grid electrode. Given that the grid width was 16 μm, the highest FOM for the Cu_2−*x*_O/Cu/Cu_2−*x*_O multilayer grid is obtained at *G* = 600 μm, with an *f*_F_ value of 0.0259. This calculated value matched the measured value, as shown in Fig. 6. The highest FOM value for the Cu_2−*x*_O/Cu/Cu_2−*x*_O multilayer grid was therefore found at *G* = 600 μm, *W* = 16 μm, and *f*_F_ = 0.025.

**Fig. 7:**
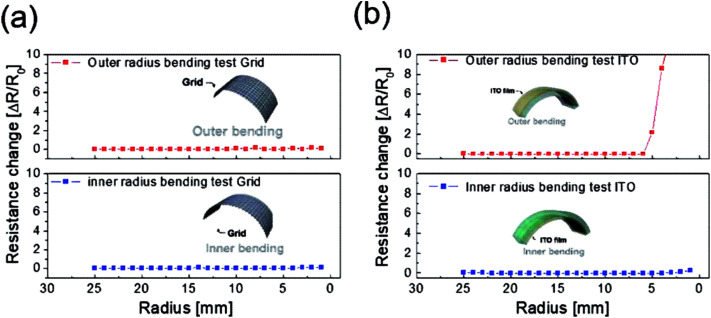
Outer and inner bending test results for (a) the Cu_2−*x*_O/Cu/Cu_2−*x*_O multilayer grid and (b) sputtered ITO electrodes fabricated on PET substrates.

To evaluate the mechanical flexibility of the optimized electrode for flexible TFHs and electrochromic devices, we measured the resistance change of the Cu_2−*x*_O/Cu/Cu_2−*x*_O multilayer grid electrode and a sputtered ITO electrode as the bending radius decreased during inner and outer bending of the substrate. Fig. 7 shows the inner/outer bending test results for both electrodes with decreasing bending radius from 25 to 2 mm. The change in resistance of the electrodes as a result of bending can be expressed as (*R* − *R*_0_)/*R*_0_, where *R*_0_ is the initial measured resistance and *R* is the resistance measured during substrate bending. The Cu_2−*x*_O/Cu/Cu_2−*x*_O multilayer grid electrode showed constant resistance until a bending radius of 2 mm, which was the limit of our bending test machine. The grid-patterned electrode therefore had a very small critical bending radius of below 2 mm due to the outstanding mechanical flexibility of the Cu interlayer. On the other hand, the sputtered ITO electrode broke at a bending radius of 5 mm in the outer bending test, while the resistance was constant for the inner bending test. As we previously reported, the resistance change is much lower during the inner bending test than during the outer bending test due to the overlapping of broken or laminated thin films.^29^

**Fig. 8:**
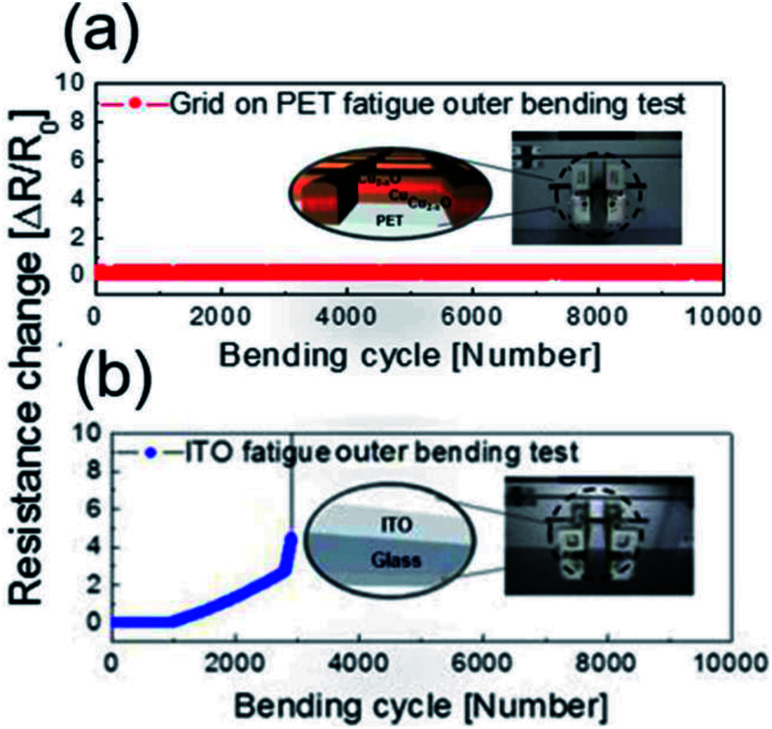
Dynamic outer bending test results for (a) Cu_2−*x*_O/Cu/Cu_2−*x*_O multilayer grid and (b) sputtered ITO electrodes for 10 000 cycles. The inset picture shows a flexed sample at a bending radius of 5 mm.

To verify the stability of the electrodes, a dynamic fatigue test was performed for the Cu_2−*x*_O/Cu/Cu_2−*x*_O multilayer grid electrode and a sputtered ITO reference electrode for 10 000 cycles, as shown in Fig. 8. Repeated outer bending was carried out at a fixed outer bending radius of 5 mm, which is a fairly small radius considering large-area smart windows with large curvature. In the case of the Cu_2−*x*_O/Cu/Cu_2−*x*_O multilayer grid electrode, the resistance was constant even after 10 000 cycles repeated bending. However, the resistance of the ITO single layer changed after 100 cycles of repeated outer bending due to crack formation and the separation of cracked ITO film. This separation led to an abrupt increase in measured resistance, as shown in Fig. 8(b). Based on the results of these tests, we confirmed that the Cu_2−*x*_O/Cu/Cu_2−*x*_O multilayer grid electrode is much more stable than an ITO single-layer electrode.

**Fig. 9:**
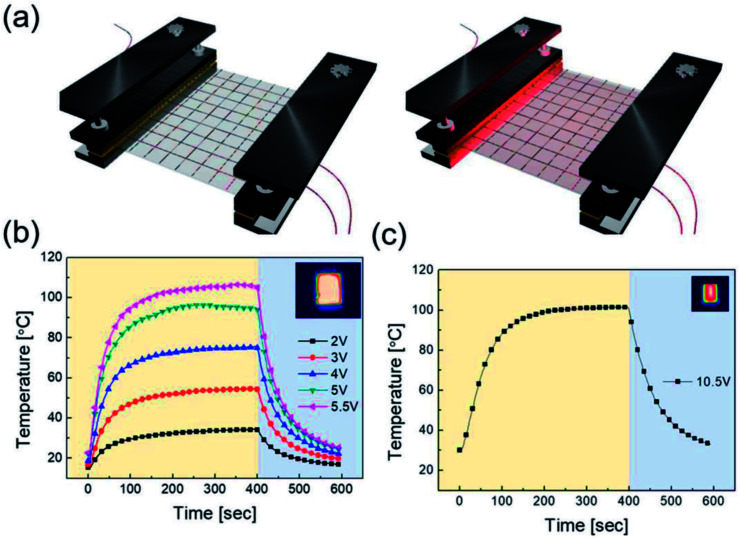
(a) Schematics of the Cu_2−*x*_O/Cu/Cu_2−*x*_O multilayer grid-based TFHs, mounted in a special jig before and after heating by applied DC power. Temperature plot of flexible TFH fabricated on (b) the Cu_2−*x*_O/Cu/Cu_2−*x*_O multilayer grid electrode as a function of applied voltage. The inset is an IR image of the flexible thin film heater at an applied voltage of 5.5 V. (c) Temperature plot of the ITO-based flexible thin film heater, reaching 100 °C at an applied voltage of 10.5 V.

To apply the RTR-fabricated Cu_2−*x*_O/Cu/Cu_2−*x*_O multilayer grid electrode to a flexible smart window, we first fabricated flexible thin film heaters on the optimized electrode, as shown in Fig. 9(a). The flexible TFHs were fabricated with a size of 25 × 25 mm^2^ using a two-terminal Ag contact configuration (Fig. S2†). A DC voltage was applied to the TFHs through the sputtered Ag metal contact electrodes at the film edge, and the temperature profiles were measured using a thermocouple placed on the surface and an IR thermometer (Fig. S3†). A flexible TFH was mounted on a specially designed sample jig to supply power and measure the temperature. Fig. 9(b) shows the temperature profiles of the TFHs with Cu_2−*x*_O/Cu/Cu_2−*x*_O multilayer grid electrodes, plotted with respect to input voltage from 2 to 5.5 V. Generally, as the input voltage increased, the saturation temperature of the TFHs increased. Due to the low sheet resistance (7.18 Ohm per square) of the Cu_2−*x*_O/Cu/Cu_2−*x*_O multilayer grid electrode, the flexible TFHs on the electrode with a width of 16 μm and pitch length of 600 μm reached a saturation temperature of 100 °C when a low DC input voltage of 5.5 V was applied. The higher saturation temperature of the flexible TFHs with Cu_2−*x*_O/Cu/Cu_2−*x*_O multilayer grid electrodes at low input voltage implies that efficient transduction of electric energy through Joule heating occurred. Based on Joule's law, the saturation temperature of the flexible TFHs can be expressed as follows.
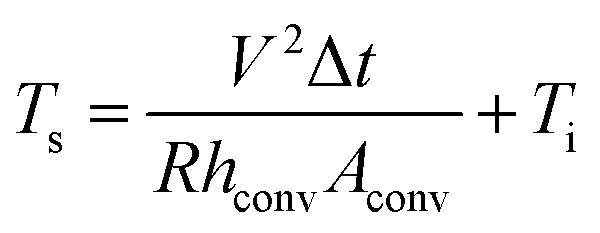


**Fig. 10:**
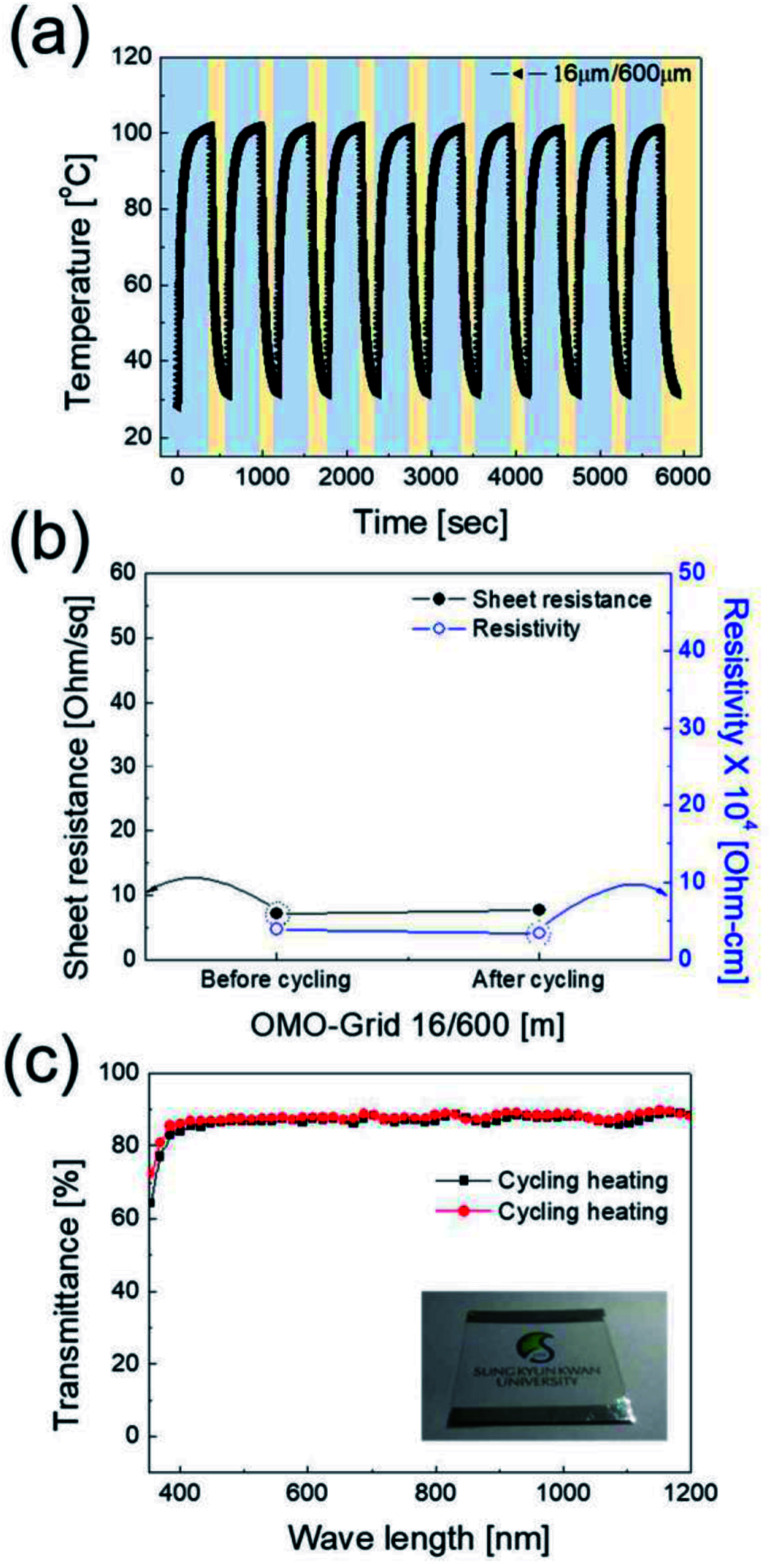
(a) Repeated heating and cooling cycles of the optimized Cu_2−*x*_O/Cu/Cu_2−*x*_O multilayer grid (600 μm grid pitch/16 μm grid width)-based thin film heater to show the stability of the Cu_2−*x*_O/Cu/Cu_2−*x*_O grid electrode. (b) Sheet resistance, resistivity, and (c) optical transmittance of Cu_2−*x*_O/Cu/Cu_2−*x*_O multilayer grid electrode on the TFHs before and after repeated 10 times heating–cooling cycles.

The power (*P*) applied to the flexible TFHs over a heating time (Δ*t*) generates heat in the TFH, as illustrated in Fig. 9(a). In the above equation, *V* is the applied voltage, *R* is the device resistance, *h*_conv_ is the heat transfer coefficient, *A*_conv_ is the surface area, and *T*_s_ and *T*_i_ are the saturation and initial temperatures. Therefore, it is apparent that the saturation temperature of flexible TFHs increases with increasing input voltage (*V*) and with decreasing resistance (*R*). Therefore, the low sheet resistance of the Cu_2−*x*_O/Cu/Cu_2−*x*_O multilayer grid electrode mainly results in the rapid attainment of a saturation temperature of 100 °C even at low voltage, which is appropriate for removing frost or deicing a smart window. On the other hand, the flexible TFH fabricated on an ITO electrode reached the saturation temperature of 100 °C at a higher input voltage of 10.5 V due to the higher sheet resistance (38.27 Ohm per square) of the sputtered ITO film. Although the ITO-based TFH did reach a temperature of 100 °C, it cannot be applied to flexed or specially shaped surfaces due to the brittleness of the sputtered ITO films. This indicates that the roll-to-roll sputtered and patterned Cu_2−*x*_O/Cu/Cu_2−*x*_O multilayer grid electrode is a promising flexible TCE for an effective transparent defroster in smart windows. It could be employed as a defogging/deicing window in automobiles, helmets, and smart windows due to its flexibility and transparency. To investigate the durability of Cu_2−*x*_O/Cu/Cu_2−*x*_O multilayer grid in the flexible TFHs, we performed repeated heating–cooling tests for 10 cycles. Fig. 10(a) shows the temperature profiles of the Cu_2−*x*_O/Cu/Cu_2−*x*_O multilayer grid-based TFHs for 10 repeated cycles. The Cu_2−*x*_O/Cu/Cu_2−*x*_O multilayer grid-based TFHs showed identical temperature profiles, rapidly reaching a saturation temperature of 100 °C when a DC voltage of 5.2 V was applied. Fig. 10(b) and (c) compared the sheet resistance and optical transmittance change before and after heating and cooling cycling test. Similar sheet resistance and optical transmittance of the Cu_2−*x*_O/Cu/Cu_2−*x*_O multilayer grid electrode indicates stability of the RTR sputtered Cu_2−*x*_O/Cu/Cu_2−*x*_O multilayer grid electrodes. Inset picture in Fig. 10(c) showed the identical transmittance of the TFH with Cu_2−*x*_O/Cu/Cu_2−*x*_O multilayer grid electrode after 10 times heating and cooling cycling.

**Fig. 11:**
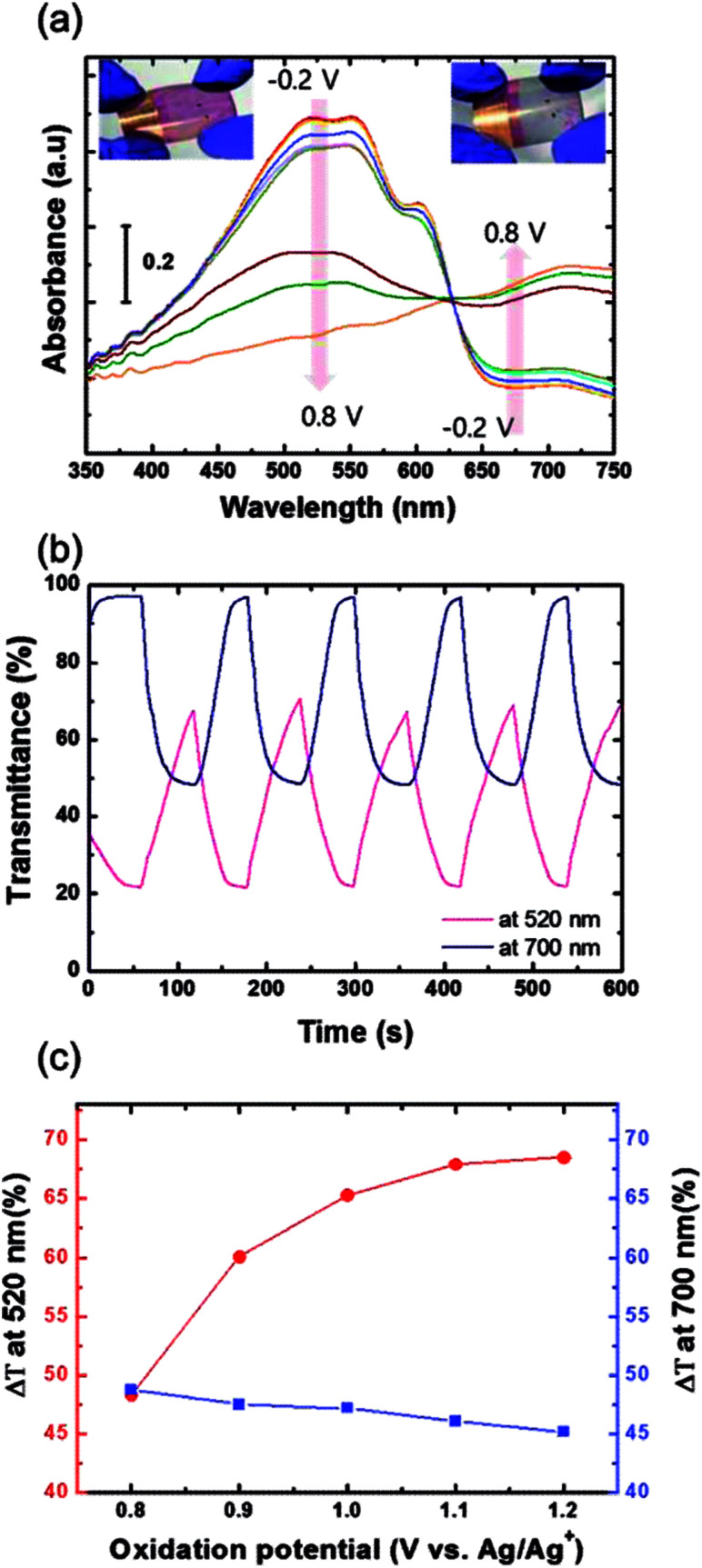
(a) Absorbance spectra for the P3HT/PEDOT:PSS film on the Cu_2−*x*_O/Cu/Cu_2−*x*_O multilayer grid electrode. The inset shows the colored and bleached states of the sample. (b) *In situ* transmittance spectra for P3HT/PEDOT:PSS film measured at 520 nm and 720 nm when a voltage pulse was applied with an interval of 60 s. (c) Change in transmittance (Δ*T*) as a function of oxidation potential.

The electrochromic absorption of the flexible P3HT/PEDOT:PSS film was monitored by UV-vis spectroscopy at different applied voltages. A series of spectroelectrochemical spectra for the sample are shown in Fig. 11(a). When a potential of from −0.2 V to 0.8 V was applied (oxidation reaction), the absorption peak at around 520 nm decreased and a new band formed around 700 nm. During this reaction, the color of the film became lighter, changing from its original red color and eventually becoming transparent blue with the formation of bipolaronic P3HT (see inset of Fig. 11(a)).^30,31^ During the reverse scan from 0.8 V to −0.2 V, the reduction reaction occurred and the P3HT films recovered their original red color. In the initial stages of the electrochemical reaction in this voltage range, the electrochromic coloration was stable without any degradation of the polymer films. However, the P3HT film coated directly on a grid substrate did not show any color change during the electrochemical reactions when a potential from −0.2 V to 0.7 V was applied, as shown in Fig. S4(a).† The abrupt decay in the absorption spectra at 0.8 V might be attributed to partial dissolution of the P3HT film due to a high electric field locally concentrated on the grid. These findings indicate that coating the Cu_2−*x*_O/Cu/Cu_2−*x*_O multilayer grid electrode with a conductive PEDOT:PSS layer homogenized the electric field in the film, and that the P3HT film was found to enable electrochemically stable optical modulation at the given potential.^32^ Although PEDOT is well known to be a cathodically coloring material with strong blue absorption,^33^ the PEDOT:PSS layer was optically inactive during electrochemical reactions between −0.2 and 0.8 V (Fig. S4(b)†), which implies that the electrochromic absorption of the P3HT/PEDOT:PSS film entirely originated from the P3HT layer. Fig. 11(b) presents the transmittance data for a P3HT film during cyclic potential switching between −0.2 V and 0.8 V. The differences in transmittance (Δ*T*) were 48.3% and 48.8% measured at 520 nm and 700 nm, respectively. When the oxidation potential was extended to 1.2 V (as shown in Fig. 11(c)), Δ*T* at 520 nm gradually increased to 68.5%, while Δ*T* at 700 nm was almost the same or slightly decreased. This means that the main absorption peak of the P3HT film located at 520 nm could be modulated by extending the oxidation potential. In particular, the bleached P3HT film under 1.2 V of oxidation potential was highly transparent, with 91.4% of transmittance (Fig. S5†). The response times during the bleaching (*τ*_b_) and coloring (*τ*_c_) processes were estimated as the time to reach 90% of the total transmittance difference. The *τ*_b_ and *τ*_c_ measured at 520 nm were 49.5 s and 30.4 s, respectively. Compared to EC devices with silver grid substrates,^34^ these values are quite reasonable with a high Δ*T*, but could be improved by further optimization of grid geometry or of the conductive PEDOT:PSS layer.

## 4. Conclusions

In summary, we developed a Cu_2−*x*_O/Cu/Cu_2−*x*_O multilayer grid electrode for TFHs and electrochromic devices using roll-to-roll sputtering and patterning processes at room temperature. The optimized Cu_2−*x*_O/Cu/Cu_2−*x*_O multilayer grid electrodes exhibited a low sheet resistance of 7.17 Ohm per square and high optical transmittance of 87.6%. In addition, we found that the Cu_2−*x*_O/Cu/Cu_2−*x*_O multilayer grid had outstanding flexibility due to the high flexibility of the Cu interlayer. The mechanical stability of the Cu_2−*x*_O/Cu/Cu_2−*x*_O multilayer grid was compared with that of a commercial ITO electrode using inner/outer bending and fatigue tests. Due to the lower sheet resistance of the electrode, the flexible TFH with the transparent Cu_2−*x*_O/Cu/Cu_2−*x*_O multilayer grid required a lower input voltage (5.5 V) to reach a saturation temperature of 100 °C than the ITO-based TFHs. Furthermore, a P3HT film coated on a Cu_2−*x*_O/Cu/Cu_2−*x*_O substrate exhibited efficient coloring/bleaching performance. The temperature profiles and EC properties indicate that the Cu_2−*x*_O/Cu/Cu_2−*x*_O multilayer grid electrode is a promising TCE for flexible smart windows.

## Conflicts of interest

The authors declare no competing financial interests.

## Supplementary Material

RA-008-C8RA03252A-s001
