# Supplementary material for: Roll-to-roll sputtered and patterned Cu2−xO/Cu/Cu2−xO multilayer grid electrode for flexible smart windows
Source: RSC Adv. 2018 Jul 30;8(47):26968–77. doi: 10.1039/c8ra03252a (PMC9083334; doi:10.1039/c8ra03252a)
Supplement: RA-008-C8RA03252A-s001 [file RA-008-C8RA03252A-s001.pdf]

Electronic Supplementary Material (ESI) for RSC Advances.
This journal is © The Royal Society of Chemistry 2018

Supplementary Information

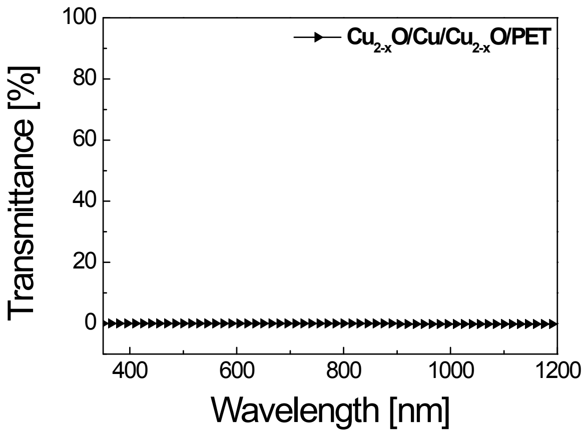

Fig. S1. Optical transmittance of as-deposited $Cu_{2-x}O/Cu/Cu_{2-x}O$ multilayer before grid patterning.

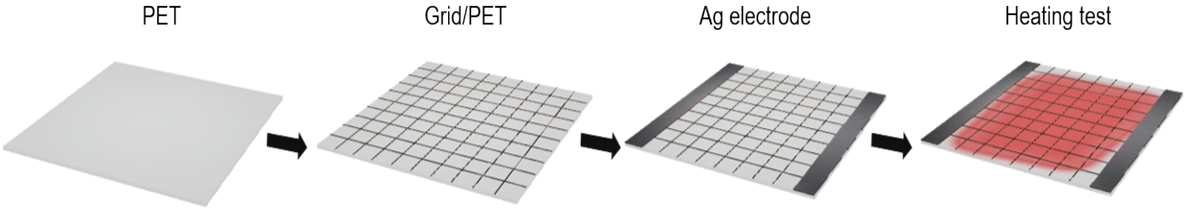

Fig. S2. Schematic of the TFH fabrication process using the RTR-sputtered $Cu_{2-x}O/Cu/Cu_{2-x}O$ multilayer grid. Two terminal-side Ag contact electrodes were fabricated on the $Cu_{2-x}O/Cu/Cu_{2-x}O$ multilayer grid films.

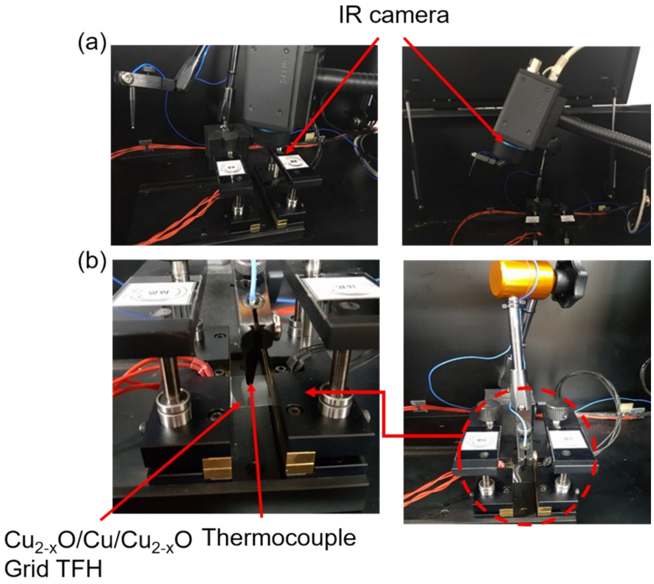

Fig. S3. Pictures of temperature measurement system for the TFHs, with (a) an IR image camera and

(b) a thermocouple.

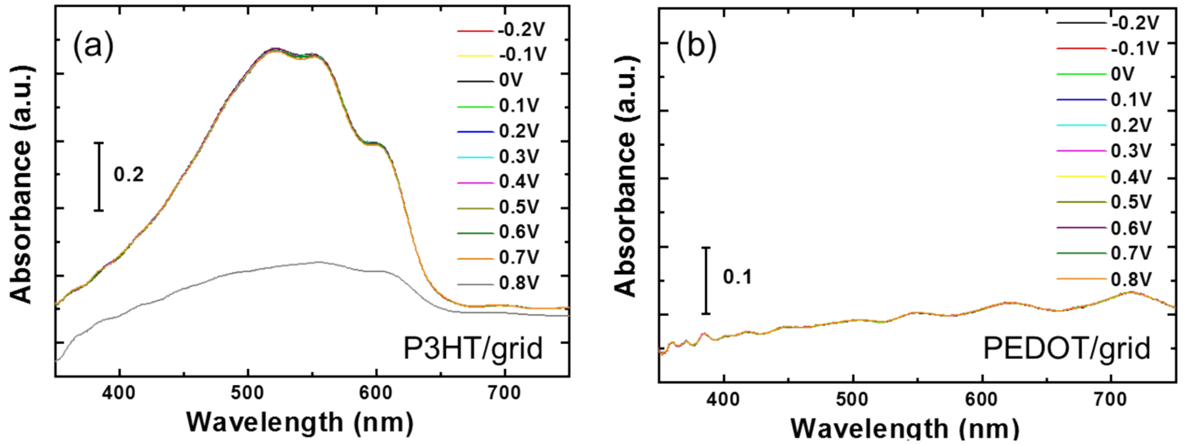

Fig. S4. Absorbance spectra obtained at potentials from -0.2 V to 0.8 V for (a) the P3HT and (b) PEDOT:PSS films spin-coated on $Cu_{2-x}O/Cu/Cu_{2-x}O$ multilayer grid electrodes.

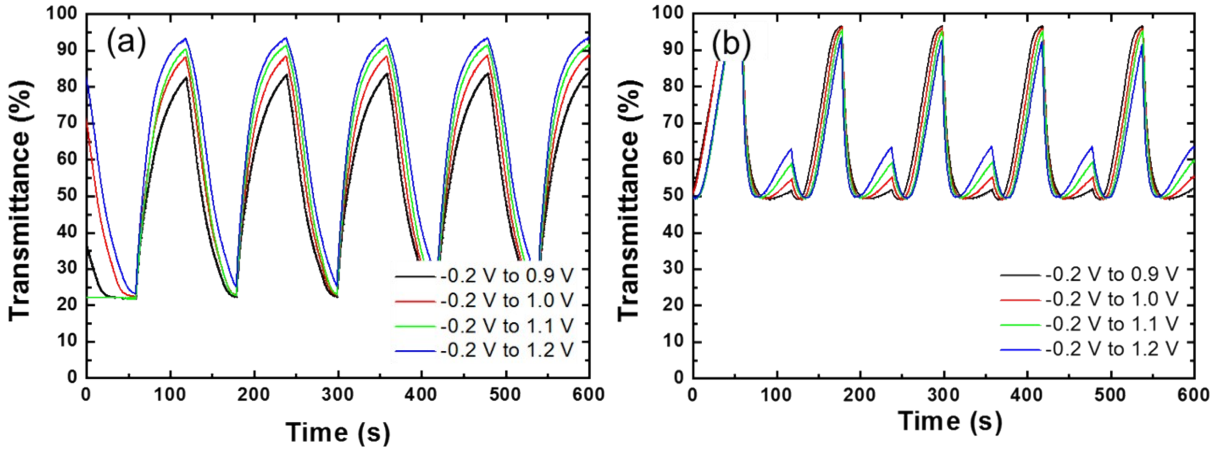

Fig. S5. *In situ* transmittance spectra for P3HT/PEDOT:PSS film measured (a) at 520 nm and (b) 720 nm when different voltage pulses were applied for an interval of 60 s.
